# Goishi tea consumption inhibits airway hyperresponsiveness in BALB/c mice

**DOI:** 10.1186/1471-2172-12-45

**Published:** 2011-08-11

**Authors:** Ryoji Hirota, Nlandu R Ngatu, Mitsuhiko Miyamura, Hiroyuki Nakamura, Narufumi Suganuma

**Affiliations:** 1 Department of Environmental Medicine, Kochi Medical School, Kochi University, Japan; 2 Department of Pharmacy, Kochi Medical School Hospital, Kochi University, Japan; 3 Department of Environmental and Preventive Medicine, Kanazawa University Graduate School of Medical Science, Japan

**Keywords:** adiponectin, allergen, airway hyperresponsiveness, eosinophil, Goishi tea

## Abstract

**Background:**

Airway hyperresponsiveness (AHR) is one of the important traits that characterize bronchial asthma. Goishi tea is a post-heating fermented tea that has been reported to have higher free radical scavenging activity. In this study, we evaluated the prophylactic effects of Goishi tea on AHR in BALB/c mice.

**Results:**

The number of inflammatory cells in BAL fluid was considerably reduced in Goishi tea/*Der f *and Gallic acid/*Der f *groups as compared with Tap water/*Der f *group. Regarding inflammatory cells in BAL, a significant reduction of eosinophils and neutrophils was observed in Goishi tea-treated mice (p < 0.01), as well as in the Gallic acid/*Der f *group (p < 0.05), as compared with Tap water/*Der f *group. In asthmatic mice (Tap water/*Der f *group), the intensity of airway resistance increased simultaneously with the increase in acetylcholine concentration in a dose-dependant way. AHR was significantly inhibited in Goishi tea/*Der f *and Gallic acid/*Der f *(p < 0.01) groups as compared with the Tap water/*Der f *group. Regarding serum specific-IgG_1_, significantly lower levels of this antibody were observed in Goishi tea/*Der f *and Gallic acid/*Der f *groups as compared with the Tap water/*Der f *group (p < 0.05). In addition, adiponectin level was significantly higher in the Goishi tea group as compared with the Tap water treated mice (p < 0.01).

**Conclusions:**

The results suggest that Goishi tea consumption exerted an inhibitory effect on eosinophilic and neutrophilic infiltration in the lung, attenuated the increase in airway resistance and increased the production of adiponectin; thus reducing Der f induced allergic inflammatory process in mice.

## Background

Airway hyperresponsiveness (AHR) is one of the important traits that characterize bronchial asthma, apart from eosinophilic infiltration, reversible airway narrowing and chronic inflammation [1,2].

Lately, there has been a growing interest in natural plants extracts containing flavonoids and polyphenols in search of new therapies thanks to their bioactive properties. Epigallocatechin gallate (EGCG) and catechin from green tea, for example, have been reported to improve cardiovascular function, increase fat oxidation in mice and exert free radicals and reactive oxygen species (ROS) scavenging activity [3-7].

Since more than 100 years ago, a post-heating fermented tea has been produced in many places in Japan. Goishi tea, known as "goishi-cha" in Japanese language, is one of a post-heating fermented tea which is produced in Otoyo town, Kochi prefecture, Japan, where local people referred to its sour taste as the tea gruel. Nowadays, most fermented tea manufacturers have already stopped the production because of lower demand; and only three of them still continue producing post-heating fermented tea in Japan.

Goishi tea is made from *Camelia sinensis *leaves, as for green tea. However, in order to make Goishi tea, two different fermentation processes are needed that are performed in two steps: the aerobic fermentation (with fungi) and the anaerobic fermentation. Green tea is processed as follows: harvested *Camellia sinensis *leaves are heated rubbed and then they are dried. Recently, traditional tea has become popular especially in Japan because of its beneficial health effects. Interestingly, Goishi tea is named "tea of legend" thanks to its efficacy in diet. Up to now, there are few publications on Goishi tea and very little is known about its bioactive properties.

We have demonstrated that Goishi tea manufacturing process improves the DPPH (2,2-diphenyl-1-picrylhydrazyl) and superoxide scavenging activity as compared with green tea [8]. The consumption of green tea has been reported to increase adiponectin expression; this chemokine is known to improve airway inflammation and some cardiovascular diseases [9,10]. Therefore, we hypothesized that Goishi tea consumption might attenuate airway inflammation. In this study, we evaluated the prophylactic effects of Goishi tea consumption in BALB/c mice induced AHR.

## Methods

### Mice

Thirty BALB/c mice, aged five weeks, were purchased from Japan SLC (Hamamatsu, Shizuoka, Japan). Animals were housed under conventional conditions at the animal facility of Kochi Medical School in filter-topped macrolon cages with a bedding of wood chips, temperature of 23°C, 50-60% relative humidity, and a 12-h light/dark cycle. They were divided into six groups of five mice each: (1) Goishi tea/*Dermatophagoides farinae*plus *(Der f)*; (2) Goishi tea/phosphate buffered saline (PBS), (3) 1% Gallic acid/*Der f*, (4) Gallic acid/PBS, (5) Tap water/*Der*, and (6) Tap water/PBS groups. They received standard lab chow ad libitum. Animals were maintained until they were 7 weeks old (~ 20-24 g body wt) at the time of sensitization. All research adhered to the animal facility guidelines of Kochi Medical School (C000144).

### Goishi tea extracts preparation

The Goishi tea sample used in this experiment was a gift from the Otoyo county office staff, Kochi prefecture, Japan. Goishi tea extract was prepared as previously described [11]. Briefly, Goishi tea is made following a traditional process; first of all, the harvested *Camellia sinensis *leaves are steamed once in a tank, then fermented on the flat plate for 7 days. Afterwards, fermented leaves are moved into another tank and then re-fermented for 10 days. Finally, the cooled leaves are cut in 2 × 2 cm pieces, then packed [8].

Goishi tea solution was made following steps; 20 g of Goishi tea dried leaves were boiled in 1000 ml distilled water at 100°C for 30 min. The extract was quickly separated from the leaves by filtration. Thus, we used the 20 mg/ml solution of Goishi tea throughout the experiment. On the other hand, 1% Gallic acid was used as a positive control. In this experiment, considering the daily intake of water by the mouse strain used (4 ml), each mouse was receiving 80 mg of Goishi tea daily for the Goishi tea/*Der f *and Goishi tea/PBS groups. Mice received tap water, Goishi and Gallic acid solutions ad libitum according to mice group from day 1 to day 37.

### Allergen and AHR induction

**Figure 1:**
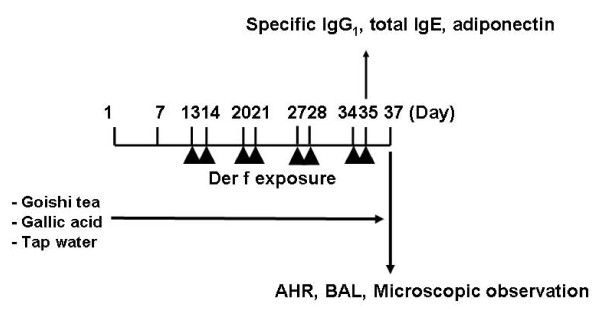
**Experimental protocol**. *Der f *: *dermatophoides farinae *; ACh: acetyl choline; AHR: airway hyperresponsiveness.

Allergen-exposed mice were actively challenged with an intratracheal instillation of 4 μg of *Dermatophagoides farinae*plus (*Der f*) plus 62.5 mg Diesel exhaust particles (DEP) solution on days 13-14-20-21-27-28-34-35, for a total of eight times as shown on the experimental protocol (Figure 1). Measurement of AHR to intravenous acetylcholine (ACh) was performed as previously described [2]. Briefly, to measure AHR, mice were anesthetized with sodium pentobarbital (60 mg/kg, i.p.) and the jugular vein was cannulated for intravenous injection of ACh. They were injected with pancuronium bromide (0.1 mg/kg, i.v.) to stop spontaneous respiration and then ventilated with a rodent ventilator (New England Medical Instruments, Inc., Medway, MA, USA). Bronchoconstriction was measured according to the overflow method, using a bronchospasm transducer (Ugo Basil 7020, Milan, Italy) connected to the tracheal cannula. Changes in respiratory overflow volume were measured using an increasing dose of ACh. The increase in respiratory overflow volume induced by ACh was represented as a percentage of the maximal overflow volume (100%) obtained by clamping the tracheal cannula. This experiment was perform with two independent experiments and, given the fact that results were similar, we included only data from the second experiment in this report.

The area under the curve (AUC) calculated from dose-response curves for ACh was used to express the magnitude of AHR. Briefly, AHR chart was saved as bmp format file; then AUC was selected and calculated using ImageJ software 1.44p (National Institutes of Health, USA) with each value of doses converted logarithmically and represented as arbitrary units.

### Collection of blood samples, measurement of total IgE, allergen-specific IgG_1 _and adiponectin in the serum

Blood samples were drawn from mice on day 35 in order to determine the level of serum IgE, allergen-specific IgG_1 _and adiponectin. Samples were kept at -80°C in the freezer until analyses were performed with the use of specific ELISA kits for mice (mouse IgE kit from Morinaga & Co. Ltd., Yokohama, Japan; mouse adiponectin kit from Otsuka Pharmaceutical CO., LTD., Tokyo, Japan). The mouse IgG_1 _kit was prepared in our laboratory (Toxicology laboratory, department of Environmental Medicine, Kochi Medical School, Kochi, Japan). Briefly, serum IgG_1 _was bound with coated *Der f *antigen, and then it was detected with horse radish peroxidase conjugated antibody. Animals were sacrificed using a high dose of pentobarbital on day 37.

### Bronchoalveolar lavage (BAL) and cells count

To perform the bronchoalveolar lavage (BAL) on day 37, animals were intraperitoneally administered 500 mg/kg of pentobarbital solution. BAL was performed with the use of 1.5 ml of saline solution and 80% (1.2 ml) of the 0.9% saline solution were recovered. Number of inflammatory cells such as eosinophils, neutrophils, lymphocytes and macrophages in the BAL fluid was recorded.

### Histopathological analysis of lung specimens

On day 37, after sacrificing animals, lung specimens from representative mice were taken and samples were fixed in 10% formalin, embedded in paraffin, sectioned at 10 μm and stained with hematoxylin and eosin (HE stain), and periodic acid-schiff (PAS stain). To examine the specimens, a light microscope (Olympus BX51, Olympus, Japan) was used at 10× magnification.

### Statistical analyses

Results were represented as the mean ± standard deviation. Statistical comparison among the treatment groups were performed by one-way ANOVA, followed by nonparametric Tukey test, with the use of SPSS software package. Results were considered to be statistically significant when p-value was less than 0.05.

## Results

### Goishi tea consumption attenuates lung inflammation

**Figure 2:**
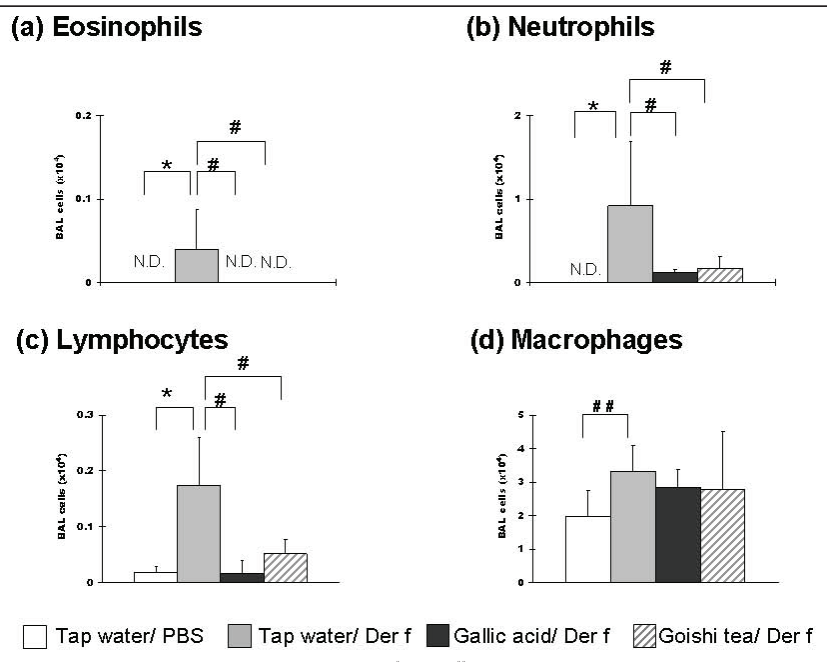
**Goishi tea consumption attenuates lung inflammation**. ^#^: p < 0.05;^##^: p < 0.01(vs. Tap water/*Der f *group). *: p < 0.05 (vs. Tap water/PBS group). p: p-value by one-way ANOVA test; BAL: bronchoalveolar lavage; *Der f*: *Dermatophagoides farinae*. The figure shows that the number of eosinophils (a), neutrophils(b) and lymphocytes (c) in BAL fluid were significantly lower in the Goishi tea/*Der f *and the Gallic acid/*Der f *groups (vs. Tap water/*Der f*; p < 0.01 and p < 0.05, respectively). Mice were intraperitoneally administered 500 mg/kg of pentobarbital solution in 1.5 ml of 0.9% saline solution, and 80% (1.2 ml) of the solution were recovered. The number of inflammatory cells was recorded. Results are mean ± SD of data from 5 mice in each group.

On day 37 of the experiment, bronchoalveolar lavage and cells count were performed for each mice group. The number of inflammatory cells in BAL fluid, which reflects airway inflammation intensity, was markedly increased in the Tap water/*Der f *group. A significantly lower number of eosinophils, neutrophils (p < 0.05) and lymphocytes (p < 0.01) was observed in Goishi tea/*Der f *mice as compared with the Tap water/*Der f *group (Figure 2). Although the number of macrophages was lower in the Goishi tea/*Der f *group than in the asthmatic mice (Tap water/*Der f *group), the difference was not significant.

### Goishi tea consumption attenuates AHR

**Figure 3:**
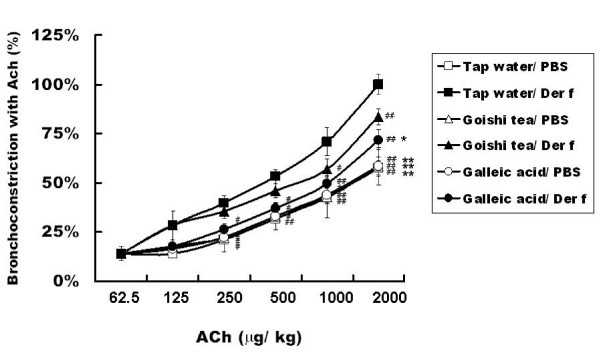
**Goishi tea consumption attenuates AHR**.^#^: p < 0.05 (vs. Tap water/*Der f *group);^##^: p < 0.01 (vs. Tap water/*Der f *group); *: p < 0.05 (vs. Goishi tea/*Der f *group). **: p < 0.01 (vs. Goishi tea/*Der f *group). AHR: airway hyper responsiveness; *Der f*: *Dermatophagoides farinae*. The figure shows that the increase in AHR, following ACh challenges, was significantly inhibited in Goishi tea/*Der f *and Gallic acid/*Der f *as compared with the Tap water/*Der f *group (p < 0.01). In addition, the AHR inhibitory effect in Gallic acid/Der f group was more efficient than that of Goishi tea/*Der f *group (p < 0.01). On the other hand, all Der f non-exposed groups showed significantly reduced airway reactivity to ACh throughout the experiment (p < 0.01, vs Tap water/Der f). To measure AHR to ACh, changes in respiratory overflow volume were measured using an increasing dose of ACh. The area under the curve (AUC) calculated from dose-response curves for ACh was used to express the magnitude of AHR. Briefly, each dose was converted logarithmically; AUC was calculated and represented as arbitrary units.

Repeated challenges to mice lung with ACh induced AHR in Tap water/Der f mice in a dose-dependent way. This process was significantly inhibited in the Goishi tea/*Der f *and Gallic acid/*Der f *groups as compared with the tap water in the Tap water/Der f group (p < 0.05) (Figure 3).

In addition, the AHR inhibitory effect in Gallic acid/Der f group was more efficient than that of Goishi tea/*Der f *group (p < 0.01).

Throughout this experiments, Gallic acid-treated mice groups and other Der f-non exposed groups showed reduced AHR as compared to Tap water/PBS group (p < 0.01), especially for the following ACh doses; 250, 500, 1000 and 2000 μg/kg.

On the other hand, Der f non-exposed groups (Goishi tea/PBS, Gallic acid/PBS, Tap water/PBS) also showed a significant AHR inhibitory effect as compared to Goishi tea/Der f group (p < 0.05) (Figure 3).

### Effect of Goishi tea consumption on serum levels of antigen-specific IgG_1 _and IgE

**Figure 4:**
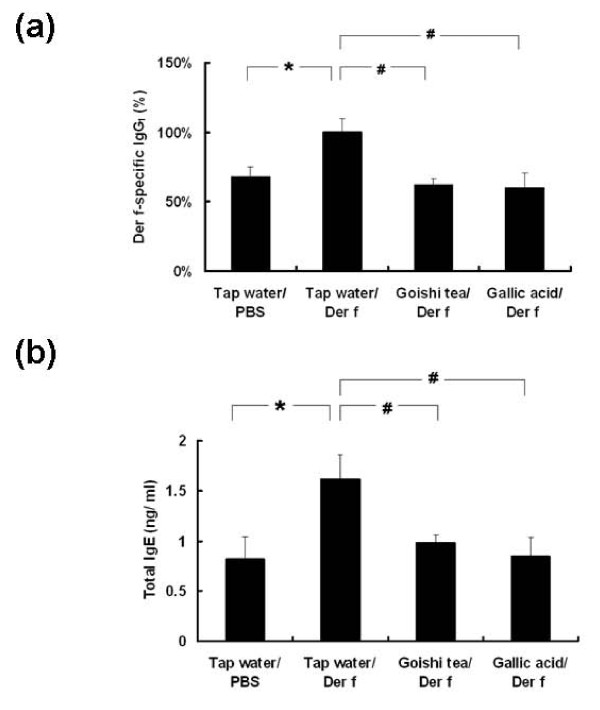
**Effect of Goishi tea consumption on serum levels of antigen-specific IgG_1 _and total IgE**. *: p < 0.05 (vs. Tap water/PBS group);^#^: p < 0.05 (vs. Tap water/*Der f *group); IgG_1_: immunoglobulin G_1_, *Der f*: *Dermatophagoides farinae*. The figure 4a shows that the serum level of *Der f *specific-IgG_1 _was significantly lower in Goishi tea/*Der f *and Gallic acid/*Der f *groups (p < 0.05) as compared with the Tap water/*Der f *group. The serum level of *Der f *specific-IgG_1 _was significantly higher in the Tap water/*Der f *group (p < 0.05) groups as compared with the Tap water/PBS group. The figure 4b shows that the serum level of total IgE was significantly lower in Goishi tea/*Der f *and Gallic acid/*Der f *groups (p < 0.05) as compared with the Tap water/*Der f *group. The serum level of total IgE was significantly higher in the Tap water/*Der f *group (p < 0.05) as compared with the Tap water/PBS group.

It is well-known that airways exposure to allergens such as *Der f *in sensitive mice species induces an increased serum level of allergen specific IgG_1_. In this experiment, significantly lower levels of *Der f *specific-IgG_1 _were observed in Goishi tea/*Der f *and Gallic acid/*Der f *(p < 0.05) groups as compared with the Tap water/*Der f *group (Figure 4a). Interestingly, there was no statistically significant difference in serum IgG_1 _levels between Goishi tea/*Der f *and Gallic acid/*Der f *groups (p > 0.05). Similarly, there was no statistically difference in terms of serum level of *Der f *specific-IgG_1 _between Goishi tea/PBS and Tap water/PBS groups, and also between Gallic acid/PBS and Tap water/PBS groups (p > 0.05).

Regarding the serum level of serum total IgE, significantly lower titers were noted in the Goishi tea/*Der f *and the Gallic acid/*Der f *groups (p < 0.01) as compared with the Tap water/*Der f *group (Figure 4b). Although lower levels of serum total IgE were also observed in Goishi tea/PBS and Gallic acid/PBS groups when compared with the Tap water/PBS group, the difference was not significant (p > 0.05). Taken together, the data suggest that Goishi tea consumption, as well as Gallic acid, exerted an immunomodulatory activity that could inhibit airway inflammation in mice.

### Effect of Goishi tea consumption on adiponectin expression

**Figure 5:**
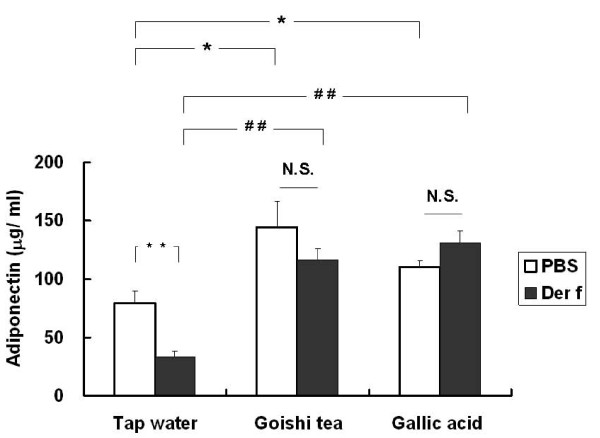
**Effect of Goishi tea consumption on adiponectin expression**. *: p < 0.05 (vs. Tap water/PBS);^##^: p < 0.01 (vs. Tap water/Der f) *Der f*: *Dermatophagoides farinae*. The figure shows that Goishi tea (p < 0.05) as well as Gallic acid (p < 0.01) consumption significantly increased adiponectin production as compared with the Tap water treated mice (p < 0.05).

Goishi tea consumption significantly increased adiponectin expression as compared with tap water-treated mice (vs. Tap water/*Der f *group; p < 0.05) (Figure 5). That was also true for gallic acid treated mice (vs. Tap water/*Der *f group; p < 0.01) as shown in Figure 5. The Goishi tea/PBS group had a relatively higher level of adiponectin as compared with the Goishi tea/*Der f *group; however, the difference was not significant (p > 0.05).

As for the trend of body weight, tap water treated mice that were exposed to the allergen had a relatively lower body weight than allergen non-exposed mice (Tap water/PBS), whereas Goishi tea consumption did not induce a significant change in the body weight as compared with the "Goishi tea/PBS" group (p > 0.05) (data not shown).

### Histological evaluation of lung specimens from Goishi tea-treated mouse and controls

**Figure 6:**
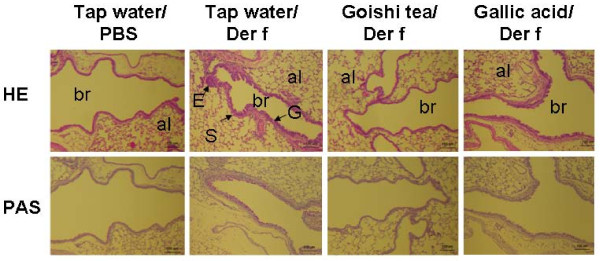
**Histological evaluation of lung specimens from Goishi tea-treated mouse and controls**. *Der f*: *Dermatophagoides farinae*; H&E: hematoxylin and eosin staining; PAS: periodic acid-schiff, al, alveolus; br, bronchiole; v, blood vessel. Scale bars = 100 μm. The figure shows a marked goblet cells hyperplasia (G), eosinophilic infiltration (E) and smooth muscle mass enlargement (S) in lung specimens from *Der f*-challenged control mice (Tap water/*Der f*), while these processes were reduced in Goishi tea and Gallic acid-treated mice groups.

After sacrificing mice using high dose of pentobarbital, full lung specimens were taken from animals for histological analysis. The hematoxylin and eosin staining of lung specimens showed a marked goblet cells hyperplasia and eosinophilic infiltration in allergen-challenged control mice (Tap water/*Der f *group), while the number of those cells was reduced in Goishi tea and Gallic acid treated mice (Goishi tea/*Der f *and Gallic acid/*Der f*) (Figure 6). Lung specimens in Goishi tea/PBS and Gallic acid/PBS showed similar to Tap water/PBS (data not shown).

## Discussion

Asthma is an escalating public health problem in children and adults; patients have an exaggerated immune response to allergens leading to lung inflammation and AHR [12], and antioxidants are thought to play a significant role in mediating the pathogenesis of asthma [13,14]. The intake of antioxidant foods could be beneficial in preventing some episodes of asthma and a number of epidemiologic studies have reported a positive association between dietary antioxidant status and lung function, and the protective effect of dietary antioxidant supplementation on asthma [15,16].

We hypothesized that Goishi tea consumption could possibly attenuate airway inflammation as it shares similar chemical components with green tea such as polyphenols, that reduce inflammatory process in injured lungs [17]. In addition, as mentioned earlier, Goishi tea contains gallic acid which inhibits pro-inflammatory cytokines' release by mast cells and upregulates IL-10 expression [10].

In this study, while an increased cellularity was observed in the allergen exposed control group (Tap water/*Der f*), there was a reduction of inflammatory cells both in lung specimens and BAL fluid in the Goishi tea/*Der f *group. In particular, Goishi tea consumption inhibited eosinophilic infiltration and goblet cells hyperplasia in mice lungs, a characteristic histological feature of airway allergic inflammation. This experiment also showed that Goishi tea consumption, as well as that of Gallic acid, significantly inhibited the expression of specific IgG_1 _and IgE in mice as compared with asthmatic mice group (Tap water/*Der f*). This suggests that Goishi tea consumption has attenuated airway inflammation in Goishi tea/*Der f *mice.

There are some foods that may increase adiponectin production [10] which has been reported to inhibit allergen-induced AHR, according to previous experimental studies [18-20]. In our experiment, we performed the measurement of this hormone in mice sera. Goishi tea has markedly increased adiponectin expression; this fact may have possibly contributed to the attenuation of AHR that was observed in the Goishi tea-treated mice groups (Goishi tea/*Der f*, Goishi tea/PBS).

This study has some limitation. The results are from animals kept in pathogen free condition and might not reflect exactly what can be found in humans who are subjects to different airway inflammation triggers. Further research in humans is needed to confirm the present results.

## Conclusions

The reduction of AHR and airway inflammation observed in Goishi tea-treated mice might result from the combination of the upregulation of adiponectin expression that it induces, its antioxidant activity and the inhibition of specific-IgG_1 _expression which were observed in this study.

Goishi tea extract inhibits airway inflammation and remodeling and has potentially beneficial effects in asthma.

## Authors' contributions

RH conceived of the study, participated in its design and coordination and wrote the manuscript, and carried out the biological studies. NN carried out the animal experiments and wrote in drafting the manuscript. MM carried out the cellular studies. HN evaluated histological data. NS performed the statistical analysis. All authors read and approved the final manuscript.
